# Bis(2-ethoxy­carbonyl­ethyl-κ^2^ *C* ^1^,*O*)(2-thioxo-1,3-dithiole-4,5-dithiol­ato-κ^2^ *S* ^4^,*S* ^5^)tin(IV)

**DOI:** 10.1107/S1600536809048971

**Published:** 2009-11-21

**Authors:** Geraldo M. de Lima, Solange M. S. V. Wardell, James L. Wardell, Edward R. T. Tiekink

**Affiliations:** a Departamento de Quimica, ICEx, Universidade Federal de Minas Gerais, 31270-901 Belo Horizonte, MG, Brazil; b CHEMSOL, 1 Harcourt Road, Aberdeen AB15 5NY, Scotland; c Department of Chemistry, University of Malaya, 50603 Kuala Lumpur, Malaysia

## Abstract

In the title compound, [Sn(C_5_H_9_O_2_)_2_(C_3_S_5_)], the immediate environment around the Sn centre is defined by two S and two C atoms that define an approximately tetra­hedral geometry. The close approach of the pendant carbonyl O atoms [Sn—O = 2.577 (3) and 2.573 (3) Å] increases the coordination number to six. Supra­molecular chains are formed along the *a* axis in the crystal structure owing to the presence of C—H⋯O contacts.

## Related literature

For original industrial inter­est in functionally substituted-alkyl-tin compounds, see: Lanigen & Weinberg (1976 ▶). For studies concerning the coordination chemistry of functionally substituted-alkyl-tin compounds, see: Harrison *et al.* (1979 ▶); Balasubramanian *et al.* (1997 ▶); Milne *et al.* (2005 ▶); Tian *et al.* (2005 ▶); de Lima *et al.* (2009 ▶). For related structures of functionally substituted-alkyl-tin compounds, see: Buchanan *et al.* (1996 ▶); Howie & Wardell, (2001 ▶). For the synthesis, see: Hutton & Oakes (1976 ▶); Valade *et al.* (1985 ▶).
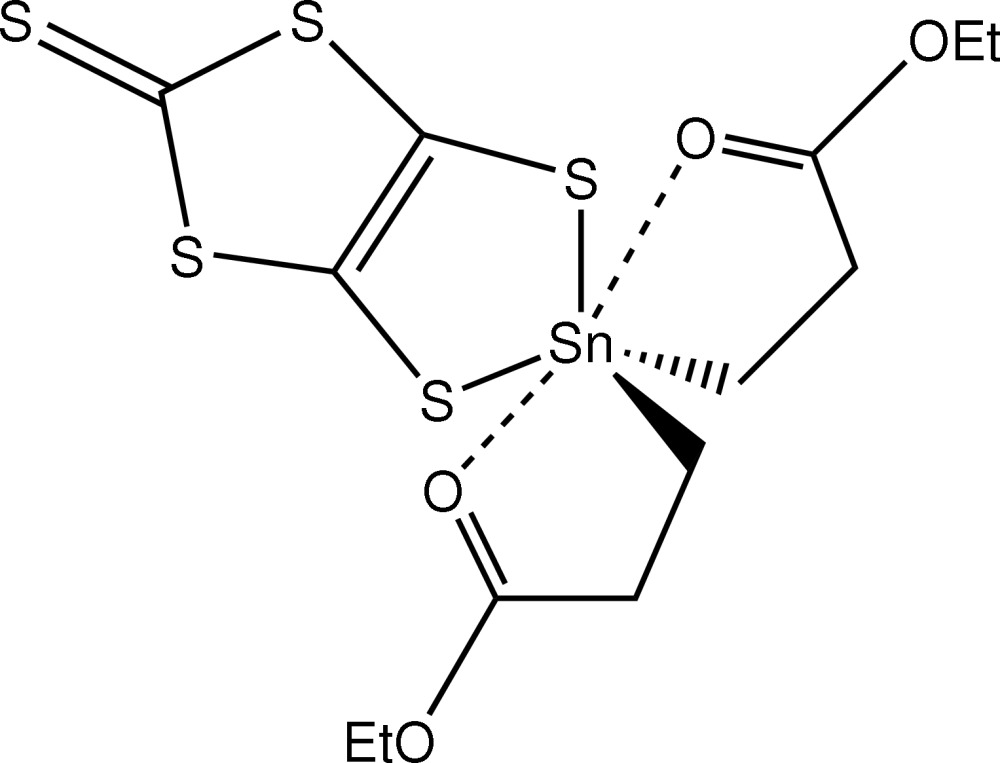


## Experimental

### 

#### Crystal data

[Sn(C_5_H_9_O_2_)_2_(C_3_S_5_)]
*M* *_r_* = 517.26
Orthorhombic, 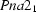

*a* = 12.1224 (2) Å
*b* = 13.3825 (2) Å
*c* = 11.9228 (2) Å
*V* = 1934.21 (5) Å^3^
*Z* = 4
Mo *K*α radiation
μ = 1.87 mm^−1^
*T* = 120 K
0.25 × 0.10 × 0.08 mm

#### Data collection

Bruker–Nonius 95mm CCD camera on κ-goniostat diffractometer
Absorption correction: multi-scan (*SADABS*; Sheldrick, 2003 ▶) *T*_min_ = 0.025, *T*_max_ = 0.052
14014 measured reflections
3895 independent reflections
3457 reflections with *I* > 2σ(*I*)
*R*_int_ = 0.056

#### Refinement

*R*[*F* ^2^ > 2σ(*F* ^2^)] = 0.029
*wR*(*F* ^2^) = 0.064
*S* = 1.04
3895 reflections
211 parameters
15 restraints
H-atom parameters constrained
Δρ_max_ = 0.64 e Å^−3^
Δρ_min_ = −0.48 e Å^−3^

### 

Data collection: *COLLECT* (Hooft, 1998 ▶); cell refinement: *DENZO* (Otwinowski & Minor, 1997 ▶) and *COLLECT*; data reduction: *DENZO* and *COLLECT*; program(s) used to solve structure: *SHELXS97* (Sheldrick, 2008 ▶); program(s) used to refine structure: *SHELXL97* (Sheldrick, 2008 ▶); molecular graphics: *DIAMOND* (Brandenburg, 2006 ▶); software used to prepare material for publication: *publCIF* (Westrip, 2009 ▶).

## Supplementary Material

Crystal structure: contains datablocks global, I. DOI: 10.1107/S1600536809048971/im2162sup1.cif

Structure factors: contains datablocks I. DOI: 10.1107/S1600536809048971/im2162Isup2.hkl

Additional supplementary materials:  crystallographic information; 3D view; checkCIF report

## Figures and Tables

**Table 1:** Hydrogen-bond geometry (Å, °)

*D*—H⋯*A*	*D*—H	H⋯*A*	*D*⋯*A*	*D*—H⋯*A*
C12—H12a⋯O1^i^	0.99	2.38	3.338 (6)	164
C7—H7a⋯O3^ii^	0.99	2.46	3.450 (7)	178

Symmetry codes: (i) 
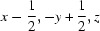
; (ii) 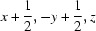.

**Fig. 1.**
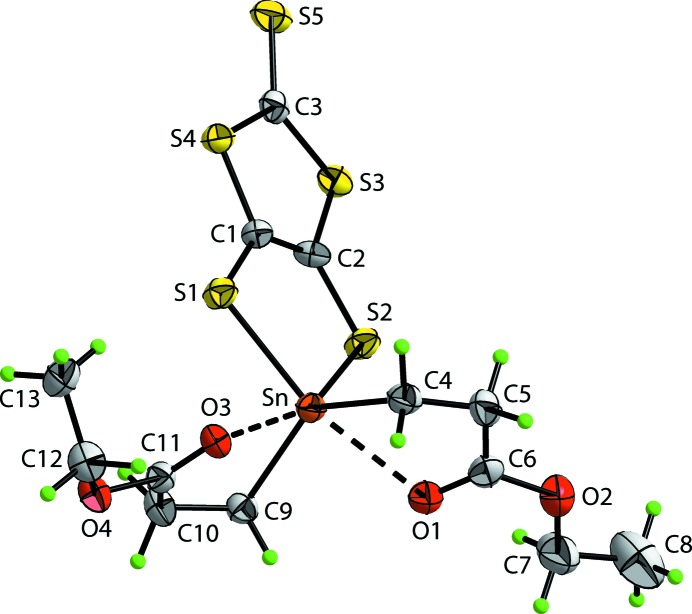
Molecular structure of (I) showing atom-labelling scheme and displacement ellipsoids at the 50% probability level.

**Fig. 2.**
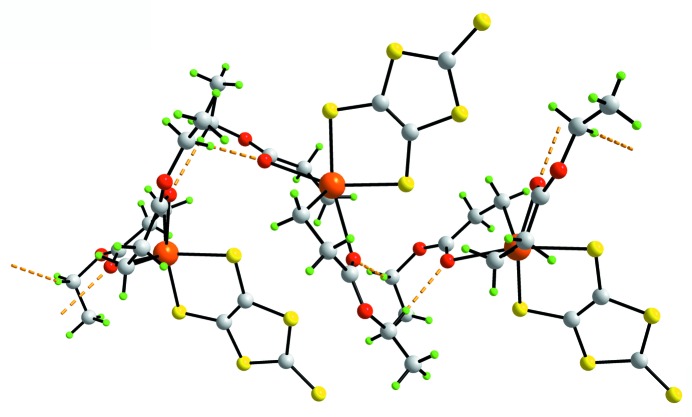
Supramolecular chain formation along the a axis in (I) mediated by C–H···O contacts (orange dashed lines).

## supplementary crystallographic information

### Comment

Functionally substituted-alkyl-tin compounds, *X*_3_SnC*R*_2_CH_2_CO_2_R' and *X*_2_Sn(C*R*_2_CH_2_CO_2_R')_2_ (*X* = halide, *R* = H or alkyl; *R*' = alkyl or aryl), are readily available from reactions first reported in the 1970's (Hutton & Oakes, 1976), starting from *R*_2_CCHCOY (Y = *R*' or OR'), HX and Sn*X*_2_ (for *X*_3_SnC*R*_2_CH_2_COY compounds) or HX and tin (for *X*_2_Sn(C*R*_2_CH_2_COY)_2_ substrates). Original interest with these compounds was primarily concerned with their industrial potential as precursors of PVC stabilizers (Lanigen & Weinberg, 1976) but also with regard to their coordination chemistry. Although the potential for use in PVC stabilization has not been realised commercially, the interest in the coordination chemistry, generally of compounds containing SnC*R*_2_CH_2_COY moieties, has been maintained over the succeeding decades. Particular interest has been paid to coordination modes of the C*R*_2_CH_2_COY ligands (de Lima *et al.* 2009; Tian *et al.*, 2005; Milne *et al.*, 2005; Harrison *et al.*, 1979). Diester-tin compounds, (MeO_2_CCH_2_CH_2_)_2_Sn*X*_2_ (*X* = halide or thiocyanate) (Balasubramanian *et al.*, 1997; Harrison *et al.*, 1979) and (MeO_2_CCH_2_CH_2_)_2_Sn(dmit) (dmit = 1,3-dithiole-2-thione-4,5-dithiolato; Buchanan *et al.*, 1996) have been shown to be molecular species with hexa-coordinate tin centres both in the solid-state and in non-coordinating solutions, as a consequence of the (*C*,*O*)-chelating ligands. Compounds (MeCOCH_2_CMe_2_)Sn*X*_2_ also contain contain hexa-co-ordinate tin centres (*X* = Cl or dmit; Howie & Wardell, 2001).

The molecular structure of (I) features a chelating dmit ligand as well as two C-bound CH_2_CH_2_CO_2_Et ligands, each of which coordinates *via* the α carbon atom. The Sn atom exists within a distorted tetrahedral C_2_S_2_ donor set, Fig. 1. Significant distortions from the ideal geometry arise from the close approach of two carbonyl-O atoms [Sn—O = 2.577 (3) and 2.573 (3) Å] thereby increasing the coordination number to six. The expanded geometry is therefore based on a highly distorted octahedron. The dmit ligand forms nearly equivalent Sn–S bond distances of 2.4805 (11) and 2.4958 (9) Å. In many respects, the molecular structure of (I) resembles that of the previously reported methyl ester analogue (Buchanan *et al.* (1996). The former has crystallographic twofold symmetry which is absent in (I) owing to a misalignment of the ethyl substituents.

In the crystal structure, molecules are connected into a supramolecular chain along the *a* axis *via* C–H···O interactions, with each molecule forming two donor and two acceptor contacts, Table 1 and Fig. 2.

### Experimental

Solutions of Cl_2_Sn(CH_2_CH_2_CO_2_Et)_2_ (0.75 g, 2 mmol) (Hutton & Oakes, 1976) in acetone (20 ml) and [NEt_4_]_2_[Zn(dmit)_2_] (0.70 g, 1 mmol) (Valade *et al.*, 1985) in acetone (20 ml) were mixed and the reaction mixture was maintained at room temperature. After 1 h, the reaction mixture was filtered and the filtrate evaporated to leave a solid residue, which after washing with water, was crystallized from acetone to give the title compound as a red-coloured crystalline solid, m.pt. 394–396 K. ^1^H NMR (CDCl_3_) δ: 1.25 [t, 3H, J(1*H*-1H) = 7.2 Hz, Me), 1.93 (t, 2H, J(^1^H-^1^H) = 7.2 Hz, J(^119^Sn-^1^H) = 84.2 Hz), CH_2_Sn), 2.96 (t, 2H, J(^1^H-^1^H) = 7.2 Hz, J(^119^Sn-^1^H) = 137.6 Hz, CH_2_CH_2_Sn), 4.20 (q, 2H, J(^1^H-^1^H) = 7.2 Hz, OCH_2_) p.p.m. ^13^C NMR (CDCl_3_, 62.9 MHz) δ: 13.9 (CH_3_), 22.8 [J(^119^Sn-^13^C) = 580 Hz, CH_2_Sn], 28.5 [J(^119^Sn-^13^C) = 46 Hz, CH_2_CH_2_Sn), 63.6 (OCH_2_), 129.8 (CC), 181.3 (CO), 210.6 (C S) p.p.m. ^119^Sn (CD_2_Cl_2_, 93.3 MHz) δ: 84.2 p.p.m. IR (KBr): 1680 (νCO), 1437 (νCC), 1031 (νCS), 890 (νC-S), 465 (νC-S) cm^-1^.

### Refinement

All H atoms were geometrically placed (C–H = 0.98–0.99 Å) and refined as riding with *U_iso_*(H) = 1.2–1.5*U_eq_*(C). Indications for disorder was found in the O2-ethyl group. Multiple sites could not be resolved, however. The O2—C7 and C7—C8 bond distances were refined with the distance restraints of 1.460 ± 0.005 Å and 1.500 ± 0.005 Å, respectively. Further, their anisotropic displacement parameters were constrained to be isotropic with the ISOR command in *SHELXL97* (Sheldrick, 2008). The structure was refined as a racemic twin precluding the determination of the absolute structure.

### Crystal data

**Table:**

[Sn(C_5_H_9_O_2_)_2_(C_3_S_5_)]	*F*(000) = 1032
*M**_r_* = 517.26	*D*_x_ = 1.776 Mg m^−^^3^
Orthorhombic, *P**n**a*2_1_	Mo *K*α radiation, λ = 0.71069 Å
Hall symbol: P 2c -2n	Cell parameters from 16139 reflections
*a* = 12.1224 (2) Å	θ = 2.9–27.5°
*b* = 13.3825 (2) Å	µ = 1.87 mm^−^^1^
*c* = 11.9228 (2) Å	*T* = 120 K
*V* = 1934.21 (5) Å^3^	Block, orange
*Z* = 4	0.25 × 0.10 × 0.08 mm

### Data collection

**Table:**

Bruker–Nonius 95mm CCD camera on κ-goniostat diffractometer	3895 independent reflections
Radiation source: Bruker-Nonius FR591 rotating anode	3457 reflections with *I* > 2σ(*I*)
graphite	*R*_int_ = 0.056
Detector resolution: 9.091 pixels mm^-1^	θ_max_ = 27.5°, θ_min_ = 3.0°
φ and ω scans	*h* = −15→14
Absorption correction: multi-scan (*SADABS*; Sheldrick, 2003)	*k* = −17→17
*T*_min_ = 0.025, *T*_max_ = 0.052	*l* = −13→15
14014 measured reflections	

### Refinement

**Table:**

Refinement on *F*^2^	Primary atom site location: structure-invariant direct methods
Least-squares matrix: full	Secondary atom site location: difference Fourier map
*R*[*F*^2^ > 2σ(*F*^2^)] = 0.029	Hydrogen site location: inferred from neighbouring sites
*wR*(*F*^2^) = 0.064	H-atom parameters constrained
*S* = 1.04	*w* = 1/[σ^2^(*F*_o_^2^) + (0.0188*P*)^2^ + 0.7375*P*] where *P* = (*F*_o_^2^ + 2*F*_c_^2^)/3
3895 reflections	(Δ/σ)_max_ = 0.002
211 parameters	Δρ_max_ = 0.64 e Å^−^^3^
15 restraints	Δρ_min_ = −0.48 e Å^−^^3^

### Special details

**Table:**

Geometry. All s.u.'s (except the s.u. in the dihedral angle between two l.s. planes) are estimated using the full covariance matrix. The cell s.u.'s are taken into account individually in the estimation of s.u.'s in distances, angles and torsion angles; correlations between s.u.'s in cell parameters are only used when they are defined by crystal symmetry. An approximate (isotropic) treatment of cell s.u.'s is used for estimating s.u.'s involving l.s. planes.
Refinement. Refinement of *F*^2^ against ALL reflections. The weighted *R*-factor *wR* and goodness of fit *S* are based on *F*^2^, conventional *R*-factors *R* are based on *F*, with *F* set to zero for negative *F*^2^. The threshold expression of *F*^2^ > 2σ(*F*^2^) is used only for calculating *R*-factors(gt) *etc*. and is not relevant to the choice of reflections for refinement. *R*-factors based on *F*^2^ are statistically about twice as large as those based on *F*, and *R*- factors based on ALL data will be even larger.

### Fractional atomic coordinates and isotropic or equivalent isotropic displacement parameters (Å^2^)

**Table:**

	*x*	*y*	*z*	*U*_iso_*/*U*_eq_	
Sn	0.529829 (17)	0.337064 (17)	0.45100 (3)	0.02754 (8)	
S1	0.53453 (8)	0.51943 (8)	0.41409 (10)	0.0335 (3)	
S2	0.73501 (8)	0.33979 (7)	0.46792 (12)	0.0345 (3)	
S3	0.88592 (8)	0.51390 (8)	0.48204 (9)	0.0346 (3)	
S4	0.72095 (7)	0.66289 (7)	0.43844 (12)	0.0321 (2)	
S5	0.94961 (10)	0.72872 (9)	0.49178 (10)	0.0453 (3)	
O1	0.5826 (3)	0.1584 (2)	0.5112 (3)	0.0379 (7)	
O2	0.6277 (3)	0.0814 (3)	0.6703 (3)	0.0573 (10)	
O3	0.3343 (2)	0.3912 (2)	0.3931 (2)	0.0309 (6)	
O4	0.2446 (2)	0.4120 (2)	0.2305 (2)	0.0360 (7)	
C1	0.6756 (3)	0.5390 (3)	0.4401 (4)	0.0295 (9)	
C2	0.7527 (3)	0.4689 (3)	0.4608 (5)	0.0307 (8)	
C3	0.8572 (3)	0.6406 (3)	0.4716 (4)	0.0313 (10)	
C4	0.4687 (4)	0.3126 (4)	0.6175 (4)	0.0356 (11)	
H4A	0.4635	0.3773	0.6572	0.043*	
H4B	0.3938	0.2834	0.6134	0.043*	
C5	0.5442 (4)	0.2423 (4)	0.6830 (4)	0.0417 (11)	
H5A	0.6080	0.2807	0.7119	0.050*	
H5B	0.5033	0.2154	0.7483	0.050*	
C6	0.5855 (4)	0.1571 (3)	0.6132 (4)	0.0412 (11)	
C7	0.6762 (6)	0.0013 (4)	0.6013 (5)	0.0691 (19)	
H7A	0.7202	0.0313	0.5399	0.083*	
H7B	0.6164	−0.0389	0.5671	0.083*	
C8	0.7474 (5)	−0.0643 (6)	0.6694 (5)	0.086 (2)	
H8A	0.8081	−0.0250	0.7012	0.129*	
H8B	0.7776	−0.1175	0.6220	0.129*	
H8C	0.7040	−0.0939	0.7302	0.129*	
C9	0.4905 (3)	0.2620 (3)	0.2973 (3)	0.0314 (10)	
H9A	0.5593	0.2486	0.2552	0.038*	
H9B	0.4553	0.1970	0.3145	0.038*	
C10	0.4131 (4)	0.3238 (3)	0.2247 (4)	0.0367 (10)	
H10A	0.4569	0.3730	0.1817	0.044*	
H10B	0.3758	0.2792	0.1704	0.044*	
C11	0.3272 (3)	0.3782 (3)	0.2923 (4)	0.0301 (9)	
C12	0.1619 (4)	0.4726 (4)	0.2870 (4)	0.0392 (11)	
H12A	0.1468	0.4441	0.3621	0.047*	
H12B	0.0924	0.4710	0.2434	0.047*	
C13	0.1992 (4)	0.5781 (3)	0.2994 (4)	0.0445 (11)	
H13A	0.2706	0.5796	0.3379	0.067*	
H13B	0.1448	0.6156	0.3433	0.067*	
H13C	0.2068	0.6086	0.2250	0.067*	

### Atomic displacement parameters (Å^2^)

**Table:**

	*U*^11^	*U*^22^	*U*^33^	*U*^12^	*U*^13^	*U*^23^
Sn	0.02877 (12)	0.02691 (13)	0.02694 (13)	−0.00144 (10)	0.00049 (14)	−0.00137 (15)
S1	0.0284 (5)	0.0272 (5)	0.0451 (7)	0.0013 (4)	−0.0039 (4)	0.0017 (4)
S2	0.0292 (4)	0.0272 (5)	0.0472 (9)	0.0016 (4)	−0.0027 (5)	0.0020 (6)
S3	0.0289 (4)	0.0381 (5)	0.0368 (7)	−0.0026 (4)	−0.0014 (4)	−0.0022 (5)
S4	0.0363 (4)	0.0271 (4)	0.0330 (7)	−0.0037 (4)	0.0001 (5)	−0.0019 (5)
S5	0.0491 (6)	0.0502 (7)	0.0367 (6)	−0.0208 (6)	0.0015 (5)	−0.0058 (6)
O1	0.0438 (18)	0.0333 (16)	0.0365 (19)	0.0018 (13)	−0.0047 (14)	−0.0023 (13)
O2	0.075 (3)	0.049 (2)	0.048 (2)	0.0012 (18)	−0.0188 (18)	0.0071 (18)
O3	0.0315 (13)	0.0359 (16)	0.0254 (16)	0.0003 (13)	−0.0035 (12)	−0.0002 (13)
O4	0.0329 (15)	0.0445 (19)	0.0306 (18)	0.0062 (14)	−0.0022 (13)	−0.0018 (15)
C1	0.0323 (17)	0.0266 (17)	0.030 (2)	−0.0050 (15)	−0.003 (2)	0.001 (2)
C2	0.0297 (16)	0.0280 (18)	0.034 (2)	−0.0046 (14)	−0.002 (2)	−0.009 (2)
C3	0.0352 (18)	0.038 (2)	0.021 (3)	−0.0066 (16)	0.0031 (17)	0.0026 (19)
C4	0.039 (3)	0.035 (3)	0.032 (3)	−0.0048 (19)	0.0015 (18)	−0.004 (2)
C5	0.051 (3)	0.047 (3)	0.027 (2)	−0.010 (2)	−0.001 (2)	0.004 (2)
C6	0.042 (3)	0.039 (3)	0.043 (3)	−0.014 (2)	−0.005 (2)	0.008 (2)
C7	0.092 (5)	0.051 (4)	0.065 (4)	0.018 (3)	−0.031 (4)	−0.016 (3)
C8	0.069 (4)	0.121 (7)	0.069 (5)	0.021 (4)	−0.012 (3)	−0.022 (4)
C9	0.034 (2)	0.031 (2)	0.029 (2)	0.0001 (19)	0.0013 (18)	−0.002 (2)
C10	0.036 (2)	0.046 (3)	0.028 (2)	0.006 (2)	−0.003 (2)	−0.003 (2)
C11	0.031 (2)	0.027 (2)	0.032 (3)	0.0007 (18)	−0.0012 (18)	0.0004 (18)
C12	0.032 (2)	0.048 (3)	0.038 (3)	0.007 (2)	0.0047 (19)	−0.002 (2)
C13	0.044 (3)	0.038 (3)	0.051 (3)	0.005 (2)	0.001 (2)	0.006 (2)

### Geometric parameters (Å, °)

**Table:**

Sn—C4	2.144 (5)	C4—H4B	0.9900
Sn—C9	2.144 (4)	C5—C6	1.498 (7)
Sn—S1	2.4805 (11)	C5—H5A	0.9900
Sn—S2	2.4958 (9)	C5—H5B	0.9900
Sn—O3	2.573 (3)	C7—C8	1.475 (4)
Sn—O1	2.577 (3)	C7—H7A	0.9900
S1—C1	1.758 (4)	C7—H7B	0.9900
S2—C2	1.743 (4)	C8—H8A	0.9800
S3—C3	1.735 (4)	C8—H8B	0.9800
S3—C2	1.742 (3)	C8—H8C	0.9800
S4—C3	1.724 (4)	C9—C10	1.520 (6)
S4—C1	1.746 (4)	C9—H9A	0.9900
S5—C3	1.644 (4)	C9—H9B	0.9900
O1—C6	1.217 (5)	C10—C11	1.505 (6)
O2—C6	1.324 (6)	C10—H10A	0.9900
O2—C7	1.474 (4)	C10—H10B	0.9900
O3—C11	1.217 (5)	C12—C13	1.490 (6)
O4—C11	1.323 (5)	C12—H12A	0.9900
O4—C12	1.454 (5)	C12—H12B	0.9900
C1—C2	1.348 (5)	C13—H13A	0.9800
C4—C5	1.527 (6)	C13—H13B	0.9800
C4—H4A	0.9900	C13—H13C	0.9800
			
C4—Sn—C9	130.01 (17)	H5A—C5—H5B	107.8
C4—Sn—S1	108.82 (14)	O1—C6—O2	122.4 (5)
C9—Sn—S1	108.33 (12)	O1—C6—C5	122.3 (4)
C4—Sn—S2	105.78 (12)	O2—C6—C5	115.2 (4)
C9—Sn—S2	107.32 (11)	O2—C7—C8	111.0 (5)
S1—Sn—S2	88.68 (3)	O2—C7—H7A	109.4
C4—Sn—O3	88.46 (13)	C8—C7—H7A	109.4
C9—Sn—O3	72.38 (13)	O2—C7—H7B	109.4
S1—Sn—O3	72.34 (7)	C8—C7—H7B	109.4
S2—Sn—O3	159.35 (7)	H7A—C7—H7B	108.0
C4—Sn—O1	71.72 (15)	C7—C8—H8A	109.5
C9—Sn—O1	81.87 (14)	C7—C8—H8B	109.5
S1—Sn—O1	163.04 (7)	H8A—C8—H8B	109.5
S2—Sn—O1	75.14 (7)	C7—C8—H8C	109.5
O3—Sn—O1	124.38 (9)	H8A—C8—H8C	109.5
C1—S1—Sn	97.92 (13)	H8B—C8—H8C	109.5
C2—S2—Sn	97.66 (11)	C10—C9—Sn	111.7 (3)
C3—S3—C2	98.14 (18)	C10—C9—H9A	109.3
C3—S4—C1	97.73 (18)	Sn—C9—H9A	109.3
C6—O1—Sn	107.4 (3)	C10—C9—H9B	109.3
C6—O2—C7	115.0 (4)	Sn—C9—H9B	109.3
C11—O3—Sn	106.9 (2)	H9A—C9—H9B	108.0
C11—O4—C12	117.1 (3)	C11—C10—C9	112.7 (4)
C2—C1—S4	116.4 (3)	C11—C10—H10A	109.1
C2—C1—S1	127.1 (3)	C9—C10—H10A	109.1
S4—C1—S1	116.5 (2)	C11—C10—H10B	109.1
C1—C2—S3	115.4 (3)	C9—C10—H10B	109.1
C1—C2—S2	127.9 (3)	H10A—C10—H10B	107.8
S3—C2—S2	116.7 (2)	O3—C11—O4	123.7 (4)
S5—C3—S4	124.2 (2)	O3—C11—C10	123.2 (4)
S5—C3—S3	123.6 (2)	O4—C11—C10	113.1 (4)
S4—C3—S3	112.2 (2)	O4—C12—C13	111.4 (4)
C5—C4—Sn	111.2 (3)	O4—C12—H12A	109.3
C5—C4—H4A	109.4	C13—C12—H12A	109.3
Sn—C4—H4A	109.4	O4—C12—H12B	109.3
C5—C4—H4B	109.4	C13—C12—H12B	109.3
Sn—C4—H4B	109.4	H12A—C12—H12B	108.0
H4A—C4—H4B	108.0	C12—C13—H13A	109.5
C6—C5—C4	112.6 (4)	C12—C13—H13B	109.5
C6—C5—H5A	109.1	H13A—C13—H13B	109.5
C4—C5—H5A	109.1	C12—C13—H13C	109.5
C6—C5—H5B	109.1	H13A—C13—H13C	109.5
C4—C5—H5B	109.1	H13B—C13—H13C	109.5
			
C4—Sn—S1—C1	99.3 (2)	Sn—S2—C2—S3	174.2 (3)
C9—Sn—S1—C1	−114.8 (2)	C1—S4—C3—S5	−176.2 (3)
S2—Sn—S1—C1	−6.96 (17)	C1—S4—C3—S3	3.4 (3)
O3—Sn—S1—C1	−178.67 (18)	C2—S3—C3—S5	176.1 (3)
O1—Sn—S1—C1	10.3 (3)	C2—S3—C3—S4	−3.5 (3)
C4—Sn—S2—C2	−102.3 (2)	C9—Sn—C4—C5	94.0 (3)
C9—Sn—S2—C2	115.7 (2)	S1—Sn—C4—C5	−129.9 (3)
S1—Sn—S2—C2	6.90 (19)	S2—Sn—C4—C5	−35.8 (3)
O3—Sn—S2—C2	29.8 (3)	O3—Sn—C4—C5	159.3 (3)
O1—Sn—S2—C2	−168.0 (2)	O1—Sn—C4—C5	32.2 (3)
C4—Sn—O1—C6	−24.6 (3)	Sn—C4—C5—C6	−40.7 (5)
C9—Sn—O1—C6	−161.6 (3)	Sn—O1—C6—O2	−168.4 (4)
S1—Sn—O1—C6	70.1 (4)	Sn—O1—C6—C5	9.8 (5)
S2—Sn—O1—C6	88.0 (3)	C7—O2—C6—O1	3.0 (7)
O3—Sn—O1—C6	−99.5 (3)	C7—O2—C6—C5	−175.4 (5)
C4—Sn—O3—C11	−156.3 (3)	C4—C5—C6—O1	17.7 (6)
C9—Sn—O3—C11	−23.1 (3)	C4—C5—C6—O2	−163.9 (4)
S1—Sn—O3—C11	93.4 (3)	C6—O2—C7—C8	164.1 (5)
S2—Sn—O3—C11	69.3 (4)	C4—Sn—C9—C10	102.9 (4)
O1—Sn—O3—C11	−89.7 (3)	S1—Sn—C9—C10	−33.2 (3)
C3—S4—C1—C2	−2.0 (4)	S2—Sn—C9—C10	−127.7 (3)
C3—S4—C1—S1	178.3 (3)	O3—Sn—C9—C10	30.6 (3)
Sn—S1—C1—C2	6.4 (5)	O1—Sn—C9—C10	160.7 (3)
Sn—S1—C1—S4	−173.9 (2)	Sn—C9—C10—C11	−38.3 (4)
S4—C1—C2—S3	−0.2 (5)	Sn—O3—C11—O4	−169.2 (3)
S1—C1—C2—S3	179.5 (3)	Sn—O3—C11—C10	9.5 (5)
S4—C1—C2—S2	−179.9 (3)	C12—O4—C11—O3	3.7 (6)
S1—C1—C2—S2	−0.2 (7)	C12—O4—C11—C10	−175.2 (4)
C3—S3—C2—C1	2.3 (4)	C9—C10—C11—O3	16.6 (6)
C3—S3—C2—S2	−178.0 (3)	C9—C10—C11—O4	−164.6 (4)
Sn—S2—C2—C1	−6.1 (5)	C11—O4—C12—C13	81.2 (5)

### Hydrogen-bond geometry (Å, °)

**Table:**

*D*—H···*A*	*D*—H	H···*A*	*D*···*A*	*D*—H···*A*
C12—H12a···O1^i^	0.99	2.38	3.338 (6)	164
C7—H7a···O3^ii^	0.99	2.46	3.450 (7)	178

Symmetry codes: (i) *x*−1/2, −*y*+1/2, *z*; (ii) *x*+1/2, −*y*+1/2, *z*.
